# Long Non-Coding RNA *GDAR* Regulates Ovine Granulosa Cells Apoptosis by Affecting the Expression of Apoptosis-Related Genes

**DOI:** 10.3390/ijms23095183

**Published:** 2022-05-06

**Authors:** Yong Wang, Yunxia Guo, Chunhui Duan, Ruochen Yang, Lechao Zhang, Yueqin Liu, Yingjie Zhang

**Affiliations:** 1 College of Animal Science and Technology, Hebei Agricultural University, Baoding 071000, China; wangyongkeyan0322@126.com (Y.W.); duanchh211@126.com (C.D.); yangruochen501@126.com (R.Y.); 18531132767@163.com (L.Z.); liuyueqin66@126.com (Y.L.); 2 College of Life Science, Hebei Agricultural University, Baoding 071000, China; gyx310@163.com

**Keywords:** granulosa cell, glucose, apoptosis, long non-coding RNA

## Abstract

Short-term dietary supplementation of ewes during the luteal phase can increase fertility, most probably by stimulating glucose uptake by the follicles. However, the molecular mechanism of glucose regulation of follicular development has not yet been clarified, especially the further study of long non-coding RNA (lncRNA) in determining fertility during follicular development. We generated granulosa cell (GC) models of different doses of glucose (0, 2.1, 4.2, 8.4, 16.8 and 33.6 mM), and observed that the highest cell viability was recorded in the 8.4 mM group and the highest apoptosis rates were recorded in the 33.6 mM group. Therefore, a control group (*n* = 3, 0 mM glucose), a low glucose group (*n* = 3, add 8.4 mM glucose), and a high glucose group (*n* = 3, add 33.6 mM glucose) of GCs were created for next whole genomic RNA sequencing. In total, 18,172 novel lncRNAs and 510 annotated lncRNAs were identified in the GCs samples. Gene Ontology indicated that differentially expressed lncRNAs associated with cell apoptosis were highly enriched. Kyoto Encyclopedia of Genes and Genomes enrichment analysis of lncRNA target genes found that the apoptosis pathway and the p53 signaling pathway were both enriched. Furthermore, we focused on the function of a lnc*GDAR* and verified that lncG*DAR* could influence cell apoptosis in GC development through affecting the mRNA and protein expression of apoptosis-related markers. These results provide the basis for further study of the lncRNA regulation mechanism in nutrition on female fertility.

## 1. Introduction

High reproductive efficiency (i.e., litter size) is the core of the sheep breeding industry. Most sheep species exhibit seasonal estrus and produce only one lamb per pregnancy. Thus, appropriate nutritional supplementation is a necessary measure to improve the reproduction of ewes. Nutritional factors have a stimulating effect on the selection of dominant follicles and follicle growth during follicle development, which have important impacts on improving lambing rate and multiple birth performance in sheep [1,2]. Granulosa cells (GCs) play an essential role in the recruitment, selection, ovulation, and atresia of follicles [3]. During normal follicle development, a mature antral follicle grows from a primordial follicle with a single oocyte surrounded by GCs. Normal proliferation and steroidogenesis of GCs are crucial for oocyte growth, maturation and fertilization, and subsequent embryonic development [4]. Therefore, elucidation of GC function is important for understanding follicular development, increasing the ovulation rate, and female fertility [5]. This will provide a scientific basis for the efficient reproductive utilization of ewes. Many studies have confirmed that short-term nutritional supplementation resulted in increased glucose concentrations in the follicular fluid microenvironment [6,7]. Our previous study showed that short-term nutritional supplementation can increase the ovulation rate by stimulating glucose uptake by the follicles [8,9]. Some human diseases, such as early follicular atresia and ovulation dysfunction caused by polycystic ovary syndrome (PCOS), are also usually accompanied by a decrease in the capability of insulin-mediated glucose uptake of ovary tissues (GCs and endometrium) [10,11]. In in vitro studies, GCs are a common germ cell model and glucose is a key substrate for GC development [12]. Normal glucose metabolism in GCs is essential for oocyte development and maturation, as well as the protection of GC development [13,14]. Chronically high glucose levels have deleterious effects on the structure and function of the ovary, especially oocytes and granulosa cells during folliculogenesis [15]. Therefore, an in-depth study of the effects of different glucose concentrations on GC development is of great significance for understanding the interaction between reproduction and nutrition.

Among the various types of non-protein coding transcripts, a class referred to as long non-coding RNAs (lncRNAs) has attracted increasing attention. lncRNAs are defined as transcripts of more than 200 nucleotides that are not translated into proteins [16]. At the post-transcriptional level, lncRNA can serve as efficient microRNA (miRNA) sponges—termed competing endogenous RNAs (ceRNAs)—that interact with miRNA to regulate gene expression [17]. Accumulating evidence shows that lncRNAs are involved in many physiological processes, including cell proliferation and apoptosis [18,19], cell development [20], and cell differentiation [21]. In reproductive activities, lncRNAs play diverse roles in the regulation of follicular development, oocyte maturation, GC differentiation and reproductive diseases. Although candidate lncRNAs related to fecundity in sheep [22] and goat [23] ovaries have been investigated, the mechanisms underlying the influence of lncRNA with respect to fecundity in ewes remain elusive. 

The proliferation and differentiation of GCs are crucial to the maturation and ovulation of follicles, and the abnormal apoptosis and degeneration of GCs are the main causes of follicular atresia [24,25]. Only 1% of the follicles eventually mature and ovulate, and >99% undergo atresia and degeneration in mammals [26]. Although GC apoptosis is known to be regulated by lncRNA *CCNL* and lncRNA *PVT1* in PCOS patients with hyperinsulinemia [27,28], a limited number of lncRNAs have been reported to be involved in glucose-stimulated GC apoptosis and follicle development. 

In this study, we hypothesized that glucose-induced GCs apoptosis could be correlated with different lncRNA and mRNA expression profiles in ovarian GCs. Hence, we investigated the differentially expressed (DE) lncRNAs and mRNAs in different glucose doses of GC groups, high glucose (33.6 mM), low glucose (8.4 mM) and control glucose (0 mM), using RNA sequencing (RNA-seq). Additionally, we further characterized the novel lncRNA, which we named granulosa-cells-differentiation-associated RNA (*GDAR*). More importantly, knockdown and overexpression of *GDAR* significantly affected GC apoptosis and apoptosis-related protein expression. Our findings will help provide insights into the regulation of reproductive nutrition. Additionally, this study may also provide a basis for identifying new therapeutic strategies for reproductive diseases, such as PCOS, which leads to ovulation failure. 

## 2. Results 

### 2.1. Model of Glucose Treatment in GCs 

**Figure 1:**
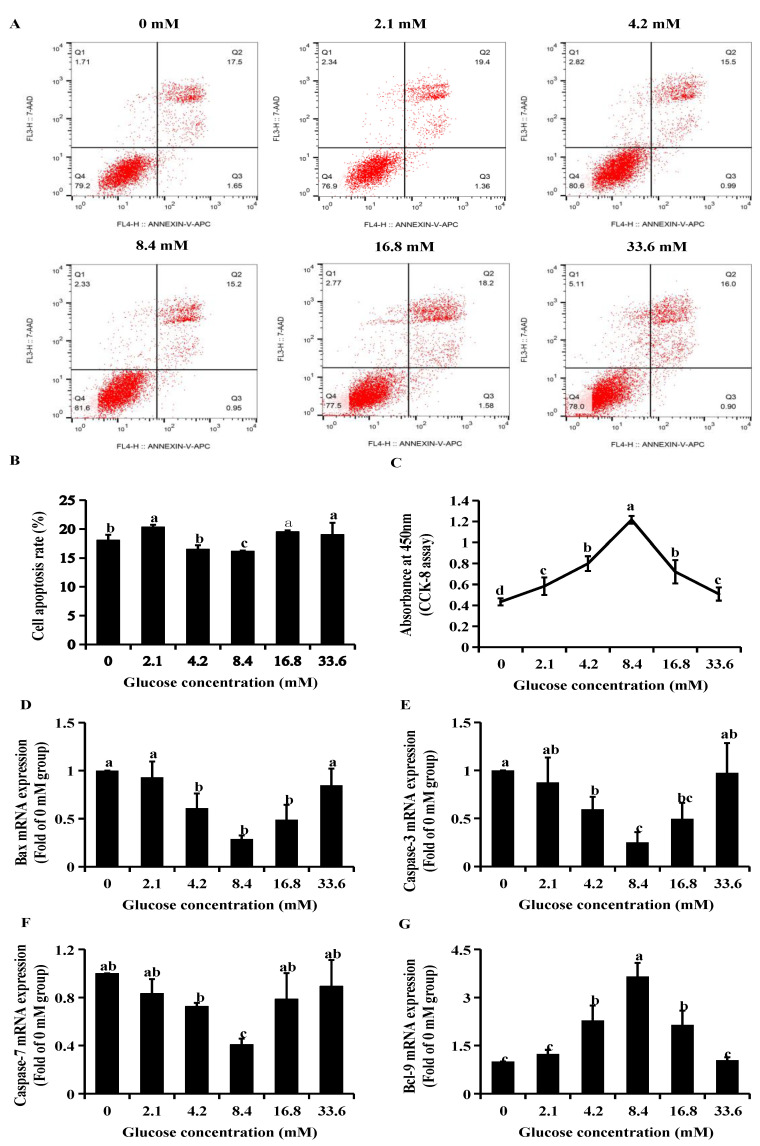

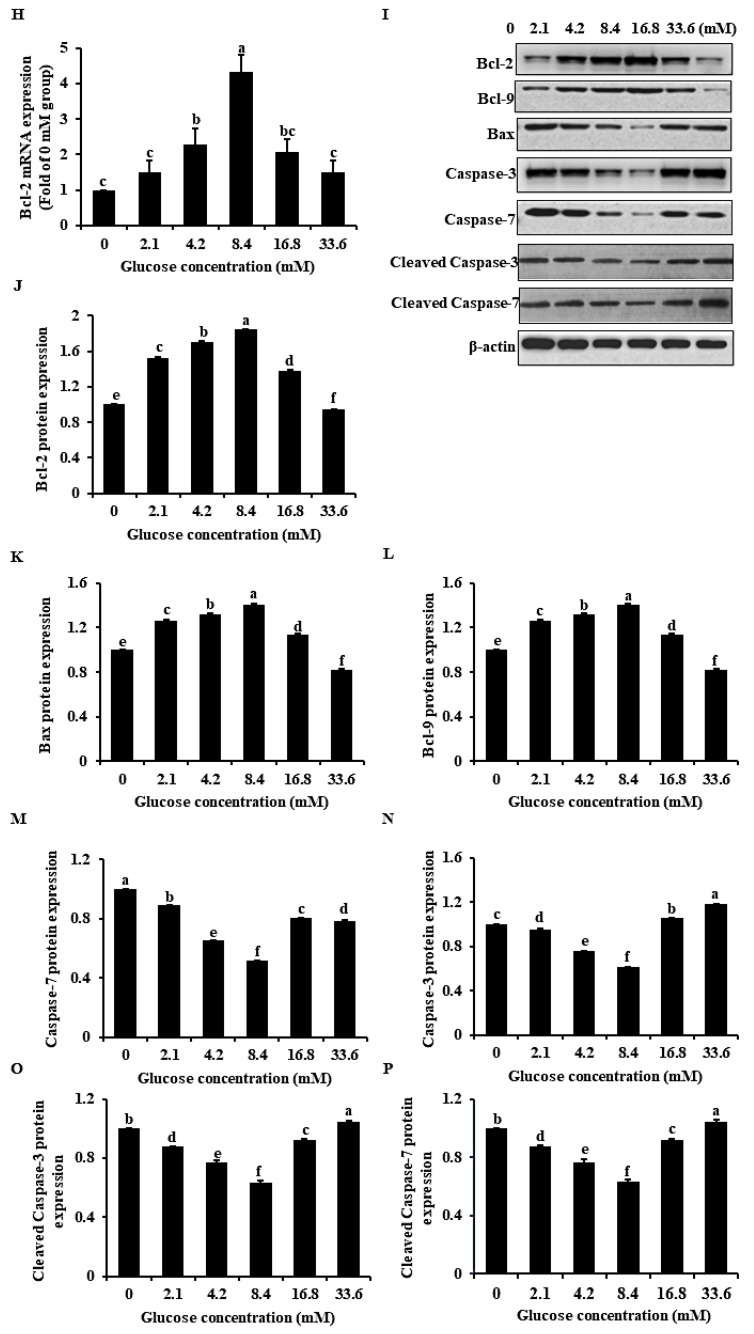
Basic characteristics of granulosa cell (GC) models in different glucose (0 mM, 2.1 mM, 4.2 mM, 8.4 mM, 16.8 mM, and 33.6 mM) groups. (**A**) Analysis of apoptosis level of GC receiving differing solutions of glucose followed by Annexin V-FITC/PIkit and flow cytometry. (**B**) Apoptosis rate of granulosa cells in different glucose concentration groups. (**C**) CCK-8 assay was performed to assess the effect of glucose on granulosa cells proliferation. (**D**–**H**) The doses of glucose affected mRNA expression of *bax*, *caspase-3*, *caspase-7*, *Bcl-9* and *Bcl-2*, which are linked to regulation of apoptosis. (**I**) The doses of glucose affected protein expression of Bcl-9, Bcl-2, bax, caspase-3 and caspase-7, which are linked to regulation of apoptosis. (**J**–**P**) The levels of Bcl-9, Bcl-2, bax, caspase-3, caspase-7, cleaved caspase-3 and cleaved caspase-7 induced by glucose were measured by western blot in GCs. Protein levels were quantified by ImageJ software and normalized to the loading controls. *n* = 3 in each group; the different lowercase letters indicate significant differences (*p* < 0.05).

The cell apoptosis assay and cell counting kit-8 (CCK-8) assay were first applied to determine the cell viability in six glucose concentration groups (0 mM, 2.1 mM, 4.2 mM, 8.4 mM, 16.8 mM, and 33.6 mM). The lowest rate of apoptosis was observed in the 8.4 mM group at 16.85 ± 0.82 (Figure 1A,B) and the highest cell proliferation occurred with the 8.4 mM group (Figure 1C). Conversely, the apoptosis rate was significantly increased in the low (0 mM) and high (33.6 mM) concentration groups, 18.05 ± 0.73 and 19.04 ± 1.42, respectively (Figure 1A,B). The results of CCK-8 analysis showed that low and high concentrations of glucose significantly inhibited the proliferation of GCs (Figure 1C). The apoptosis-related mRNAs (*caspase-3*, *caspase-7*, and *bax*) were down-regulated (Figure 1D–F), and the anti-apoptosis-related mRNAs (*bcl-2* and *bcl-9*) were up-regulated in the 8.4 mM groups compared to other groups (Figure 1G,H). Furthermore, our results showed that apoptosis related-proteins (bax, caspase-3, caspase-7, cleaved caspase-3 and cleaved caspase-7) expression levels were decreased and antiapoptotic proteins (Bcl-2 and Bcl-9) expression levels were increased in 8.4 mM glucose groups compared with other groups (Figure 1I–P). Conversely, the low (0 mM) and high (33.6 mM) glucose treatments significantly upregulated the expression of apoptosis-related proteins and downregulated the expression of antiapoptotic-related proteins (Figure 1I–P). Together, these data suggest that glucose regulates GCs differentiation and that the proliferation rates were promoted in the 8.4 mM glucose group and inhibited in the low (0 mM) and high (33.6 mM) glucose groups. The 8.4 mM group represents the optimum concentration for glucose metabolism in media used for the culture of ovine GCs. Therefore, we chose three solutions of GCs for the next RNA-seq, respectively: control (glucose concentration = 0 mM), low group (glucose concentration = 8.4 mM), and high group (glucose concentration = 33.6 mM).

### 2.2. RNA Sequencing Identified the Features of lncRNA and mRNA in GCs

**Figure 2:**
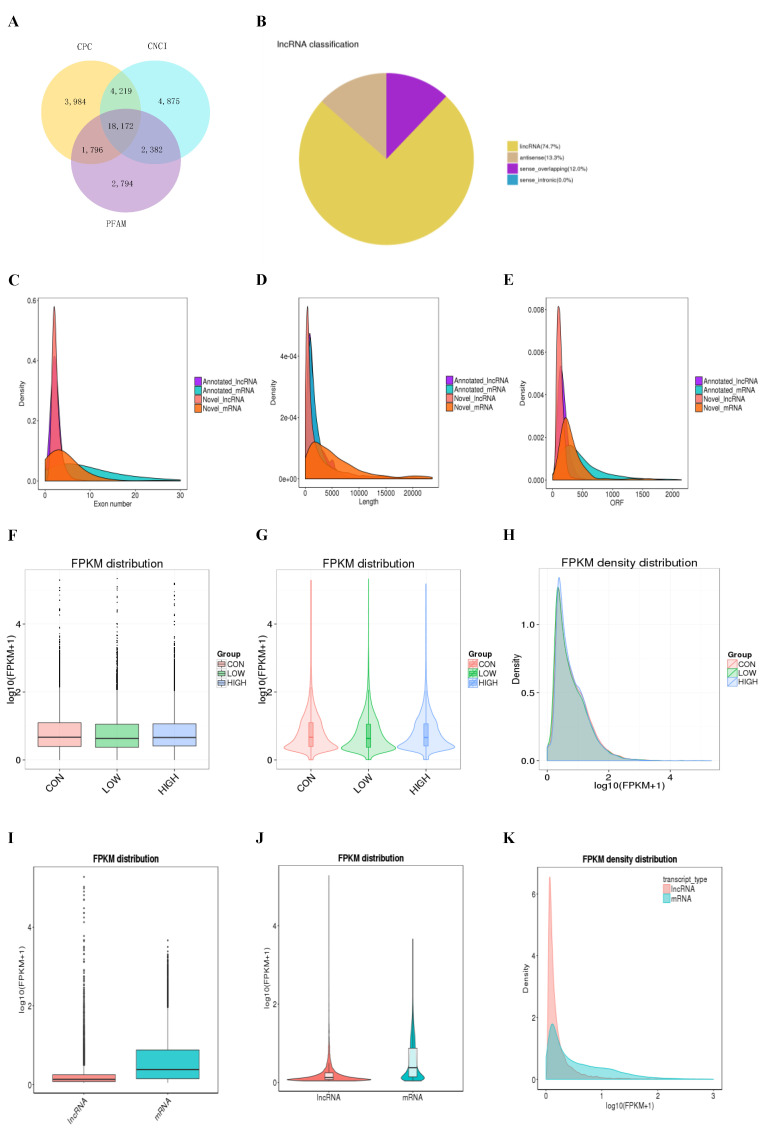
Characterization of long non-coding RNA (lncRNA) and mRNA in GCs. (**A**) Coding potential analysis via CNCI (Coding-Non-Coding Index), CPC (Coding Potential Calculator), PFAM (Protein Families database). Those sequences simultaneously shared by the above three tools were selected as candidate lncRNAs. (**B**) The classification of identified lncRNAs. (**C**–**E**) Density distribution diagram showing the expression features of exon number (**C**), length (**D**), and opening reading frame (ORF) (**E**) of annotated lncRNAs, novel lncRNAs, and mRNAs in GCs. (**F**–**H**) Boxplot (**F**), violin plot (**G**), and density distribution diagram (**H**) showing the expression features of GCs from in different glucose treatment groups. (**I**–**K**) Boxplot (**I**), violin plot (**J**), and density distribution diagram (**K**) showing the expression features of lncRNA and mRNA in ovary GCs. FPKM, fragments per kilobase million.

Three levels of glucose were added to the medium (Gibco, Carlsbad, CA, USA, Cat No. 11966025) in which GCs were cultured in vitro, GC samples were collected after 24 h of culture. RNA sequencing (RNA-seq) data was analyzed from GC samples, in which 98,057,424-155,947,936 raw data and 96,829,522-154,249,698 clean data were obtained (Table 1). Using this method, 18,172 novel lncRNAs were identified from an intersection of the analysis results of the Coding Potential Calculator (CPC), Coding-Non-Coding Index (CNCI), and Protein Families database (PFAM) (Figure 2A), which included 13,574 lncRNAs (74.7%), 2,417 antisense lncRNAs (13.3%), and 2,180 overlapping lncRNAs (12.0%) (Figure 2B). We compared the number of overlapping mRNAs in the low, high, and control groups and found that annotated and novel lncRNAs were smaller in size, had fewer exons, and fewer open reading frames than mRNAs (Figure 2C–E). There was no significant difference in transcript levels in GCs from the low, high, and control groups (Figure 2F–H). However, the transcript levels of mRNA were significantly higher than levels of lncRNAs in the GC samples (Figure 2I–K).

### 2.3. Differential Expression and Cluster Analysis of lncRNAs and mRNAs

**Figure 3:**
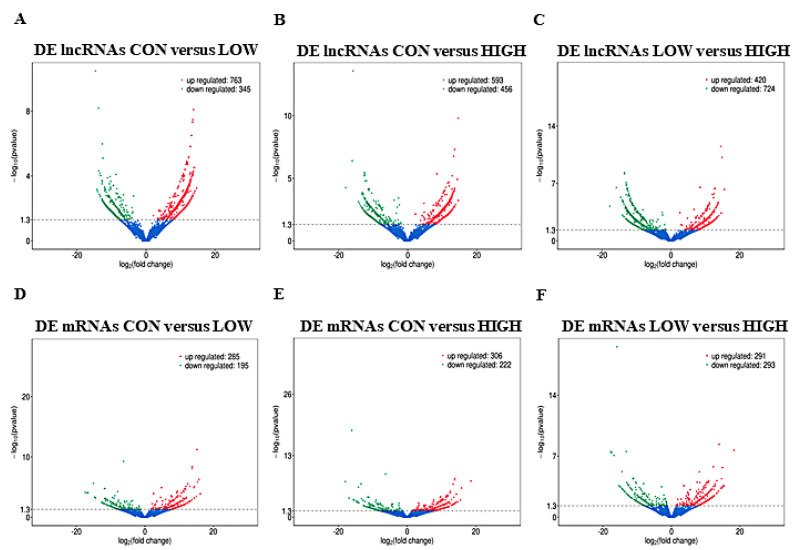

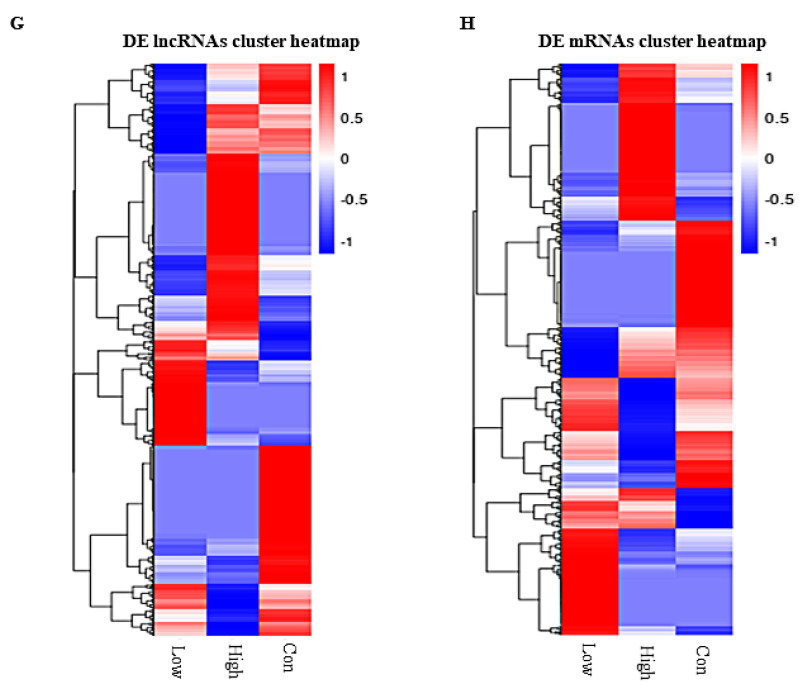
The expression profiling changes of lncRNAs and mRNAs in GCs. (**A**–**C**) Volcano plot indicating up− and downregulated lncRNAs of ovary GCs in different glucose treatment groups (CON versus LOW, CON versus HIGH and LOW versus HIGH); up− and downregulated genes are colored in red and green, respectively. (**D**–**F**) Volcano plot indicating up− and downregulated mRNAs of ovary GCs in different glucose treatment groups (CON versus LOW, CON versus HIGH and LOW versus HIGH); up− and downregulated genes are colored in red and green, respectively. (**G**,**H**) Heatmap of lncRNAs (**G**) and mRNAs (**H**) showing hierarchical clustering of changed lncRNAs and mRNAs of GCs in different glucose treatment groups; up− and downregulated genes are colored in red and blue, respectively.

There were 1108 (763 upregulated and 345 downregulated), 1049 (593 upregulated and 456 downregulated), and 1144 (420 upregulated and 724 downregulated) DE lncRNAs identified in the CON versus LOW (Figure 3A; Table S3), CON versus HIGH (Figure 3B; Table S4), and LOW versus HIGH groups (Figure 3C; Table S5), respectively. There were 480 (285 upregulated and 195 downregulated), 528 (306 upregulated and 222 downregulated), and 584 (291 upregulated and 293 downregulated) DE mRNAs identified in the CON versus LOW (Figure 3D; Table S6), CON versus HIGH (Figure 3E; Table S7), and LOW versus HIGH groups (Figure 3F; Table S8), respectively. Hierarchical clustering of the DE lncRNAs (Figure 3G) and DE mRNAs (Figure 3H) revealed the expression patterns of the individuals for the same three comparisons.

### 2.4. Systematic Functional Analysis of Differentially Expressed lncRNAs and mRNAs

**Figure 4:**
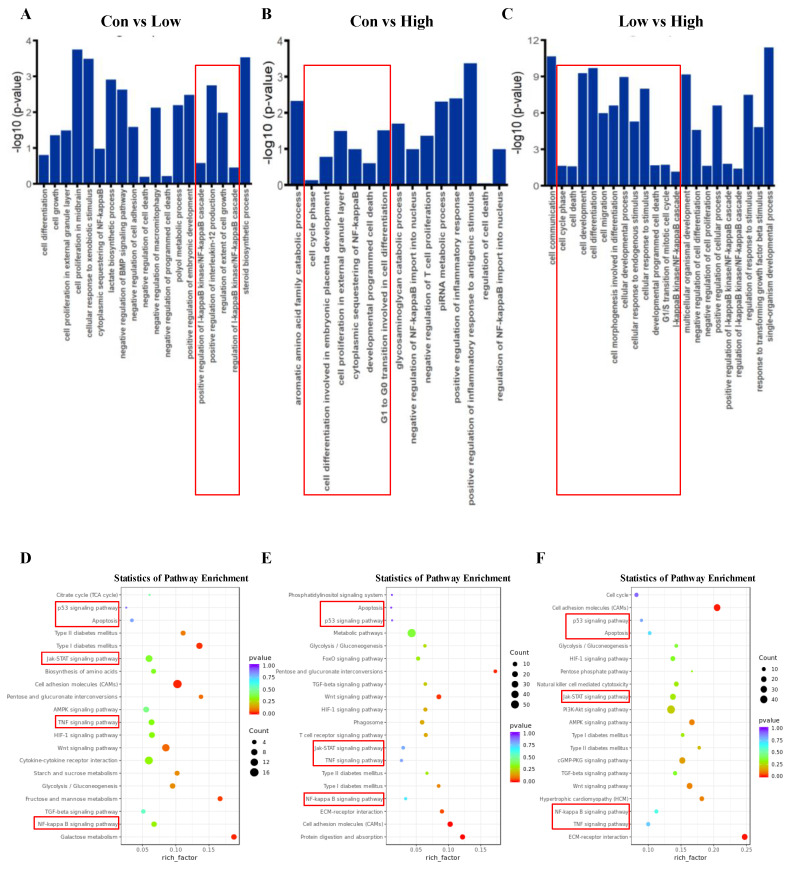
Gene Ontology (GO) and Kyoto Encyclopedia of Genes and Genomes (KEGG) Analyses of differentially expressed lncRNA target genes and mRNAs in GCs in different glucose treatment groups. (**A**–**C**) GO categories (biological process) of differential lncRNA target genes in different glucose treatment groups (CON versus LOW, CON versus HIGH and LOW versus HIGH). (**D**–**F**) KEGG analysis of differential lncRNA target genes in different glucose treatment groups. The size and color of each bubble represents the number of genes in each pathway and *p* value, respectively.

To determine the possible functional significance of observed changes in lncRNA levels in the high and low glucose-induced GCs groups and the control group, a Gene Ontology (GO) term enrichment analysis was performed. There were 21,637 background genes in total. The paper summarizes the significantly enriched GO terms of lncRNAs regarding biological process (Figure 4A–C), cellular component (Figure S1), and molecular function (Figure S2), respectively. Interestingly, the differentially expressed lncRNAs were found to be similar and significantly associated with cell development in biological process term enrichment (Figure 4A–C). It is noteworthy that cell death and nuclear factor kappa B (NF-Kappa B) cascade were enriched among the three comparison groups, especially in the low and high groups, suggesting that cell apoptosis and cell death might play a critical role in glucose-induced GCs differentiation. 

In addition, the enrichment of cellular components and molecular function also revealed a similar pattern. For example, cell periphery, extracellular space, and extracellular matrix were all obviously enriched in lncRNA levels in low and high groups (Figure S1). For molecular function enrichment, terms regarding binding, such as protein binding and enzyme binding, were enriched, which suggested that complex physiological processes are involved in glucose-induced GC differentiation (Figure S2).

To determine if there were some specific pathways changed, Kyoto Encyclopedia of Genes and Genomes (KEGG) enrichment analysis in lncRNA target genes was performed. Notably, the apoptosis pathway and p53 signaling pathway were both enriched in three comparison groups (Figure 4D–F); this finding is consistent with the GO term enrichment results. The tumor necrosis factor (TNF) signaling pathway [29], NF-Kappa B signaling pathway [30,31], and Janus kinase (JAK)-signal transducer and activator of transcription (STAT) signaling pathway [32], were enriched, which previous studies have implicated in cell apoptosis; this paper’s KEGG enrichment analysis also suggests their significance.

### 2.5. Validation of DE lncRNAs by Quantitative Real-Time PCR (qRT-PCR)

**Figure 5:**
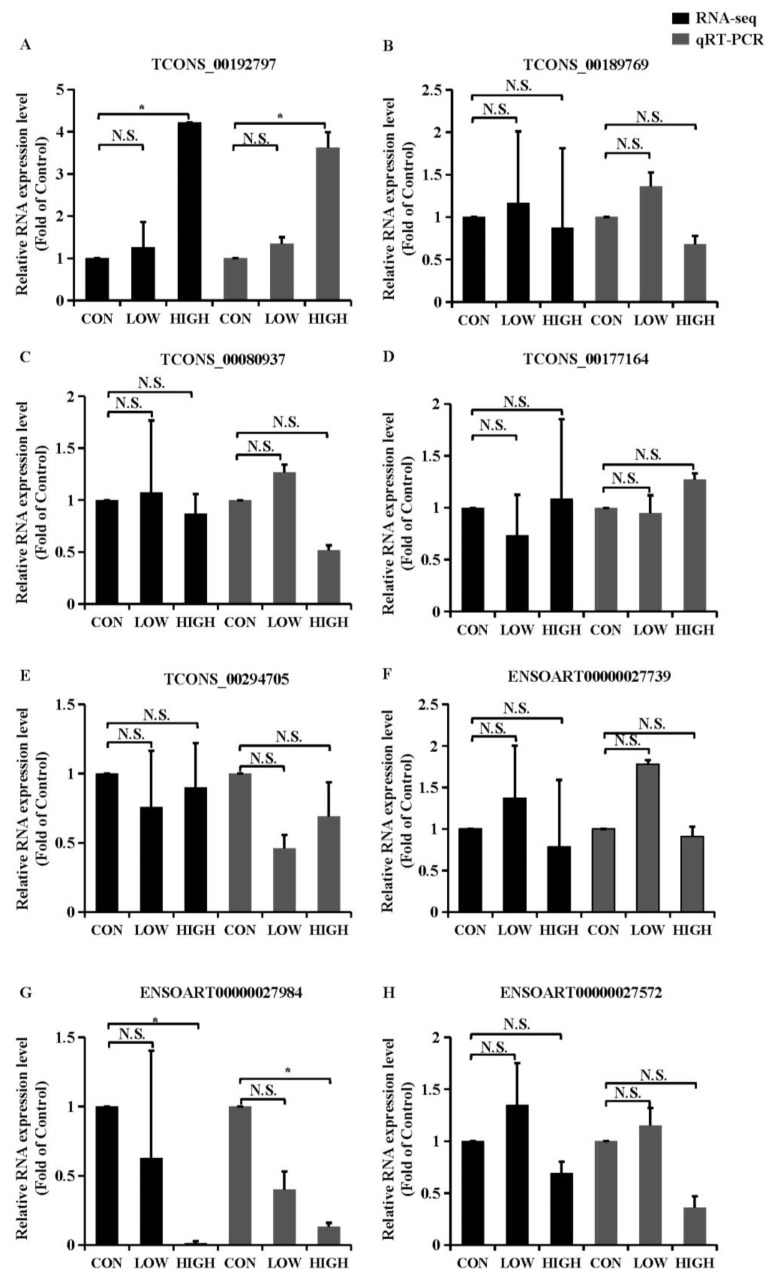
Quantitative real-time PCR (qRT-PCR) validations. (**A–H**) qRT-PCR validations of eight regulated lncRNAs in GCs from in different glucose treatment groups. Values represent means ± SEM for three individuals. * *p* < 0.05. N.S., not significant.

To validated the reliability of the sequencing results and to provide the basis for further study, eight DE lncRNAs (*TCONS_00192797*, *TCONS_00189769*, *TCONS_00080937*, *TCONS_00177164*, *TCONS_00294705*, *ENSOARG00000027739*, *ENSOARG00000027984* and *ENSOARG00000027572*) that have targeting relationships with apoptosis-related genes were chosen to analyze by Quantitative real-time PCR (qRT-PCR) (Figure 5; Table S9). All lncRNAs selected for validation were statistically significant and consistent with the results obtained from next-generation sequencing, which indicated the high quality and validity of the RNA-seq.

### 2.6. Downregulation of lncRNA TCONS_00128966 in GCs Is related to apoptosis

**Figure 6:**
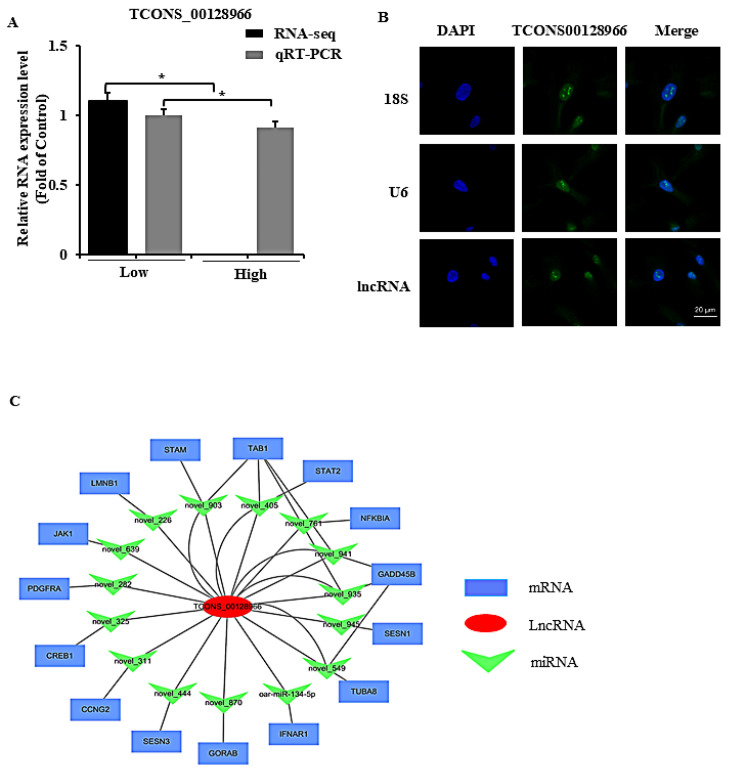
Downregulation of lnc*GDAR* in GCs is related to apoptosis. (**A**) The expression of lnc*GDAR* detected by qRT-PCR. (**B**) Expression location of lnc*GDAR* in GCs by fluorescence in situ hybridization (FISH), lnc*GDAR* was labeled with green fluorescence, and the nuclei were stained by DAPI (blue). Scale bar: 20 μm. (**C**) LncRNA-miRNA-mRNA interaction network consisting of 1 lncRNAs (red circles), 15 miRNAs (green arrows), and 15 mRNAs (green squares). lncRNA, long non-coding RNA; miRNA, microRNA; mRNA, messenger RNA. Values represent means ± SEM for three individuals. * *p* < 0.05.

By considering GO terms, KEGG pathway enrichment, and our findings using the glucose model, we found that apoptosis could be a critical factor in glucose-induced abnormal GCs function, especially under high glucose conditions (i.e., 33.6 mM). Among these differentially expressed lncRNAs, a lncRNA *TCONS_00128966* that was significantly downregulated in high glucose groups, and the analysis of qRT-PCR also verified this result (Figure 6A). The intergenic lncRNA *TCONS_00128966* is located on chromosome chr18:14283616–14290522, and its RNA sequence is shown in Figure S3. The lncRNA *TCONS_00128966* binding miRNAs were predicted as candidates using RNAhybrid and miRanda software. A ceRNA (*TCONS_00128966*-miRNA-mRNA) network with 15 miRNAs and 15 mRNAs was constructed in GCs and belongs to the apoptosis-related signaling pathway (Figure 6B, Table S10). Thus, we suspected that lncRNA *TCONS_001289* plays an important role in GCs development and hereinafter refer to this lncRNA as lnc*GDAR* for convenience. Fluorescence in situ hybridization (FISH) revealed that lnc*GDAR* could be expressed in both the nucleus and cytoplasm (Figure 6C).

### 2.7. lncGDAR Inhibits Apoptosis of GCs

**Figure 7:**
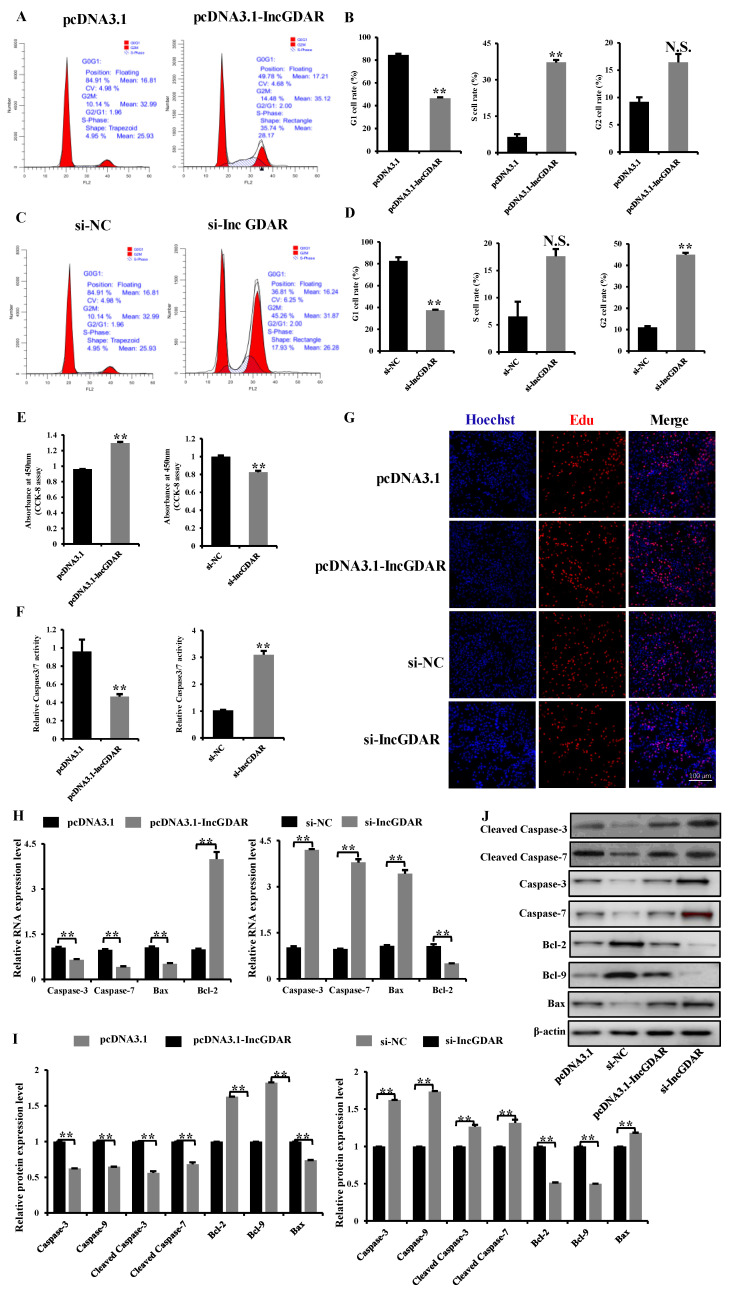
Mechanism study of lnc*GDAR* regulation of GC apoptosis. (**A**,**B**) Cell cycle analysis of GCs at 48 h after transfection of pcDNA3.1-lnc*GDAR* and pcDNA3.1 empty plasmids. (**C**,**D**) Cell cycle analysis of GCs at 48 h after transfection of si-lnc*GDAR* and si-NC. (**E**) CCK-8 assay was performed to assess the effect of lnc*GDAR* overexpression and knockdown on GCs proliferation. (**F**) Caspase3/7 activity assay after transfection of pcDNA3.1-lnc*GDAR* and pcDNA3.1 empty plasmids, or si- lnc*GDAR* and si-NC in GCs. (**G**) 5-Ethynyl-2′- deoxyuridine analysis after transfection of pcDNA3.1-lnc*GDAR* and pcDNA3.1 empty plasmids, or si-lnc*GDAR* and si-NC in proliferating GCs; scale bars are 100 μm. (**H**) qRT-PCR of relative expression of apoptosisi-related mRNA in GCs transfected with pcDNA3.1-lnc*GDAR* and in GCs transfected with si-lnc*GDAR*. (**I**,**J**) Overexpression and knockdown of lnc*GDAR* affected the expression of apoptosis-related proteins (caspase-3, caspase-7, cleaved caspase-3, cleaved caspase-7, bax, Bcl-2 and Bcl-9). Values represent means ± SEM for three individuals. **, *p* < 0.01. N.S., not significant.

To elucidate the potential role of lnc*GDAR* in apoptosis, the cell cycle and caspase3/7 activity assay were first detected in four groups: a positive control group (pcDNA3.1), an overexpression group (pcDNA3.1-Inc*GDAR*), a negative control group (si-NC), and a knockdown group (si-Inc*GDAR*). Overexpression of lnc*GDAR* reduced the number of cells that progressed to the G0/G1 phase and increased the number of cells that progressed to the S phase (Figure 7A,B), and significantly reduced caspase3/7 activity (Figure 7E). Conversely, lncG*DAR* knockdown increased cell cycle arrest in the G0/G1 phase (Figure 7C,D) and significantly promoted GC caspase3/7 activity (Figure 7F). To determine the proliferation ability in four groups, CCK-8 was performed; we found lnc*GDAR* overexpression significantly increased the proliferation rate in GCs (Figure 7G). Conversely, proliferation was significantly inhibited after lnc*GDAR* knockdown (Figure 7H). 5-Ethynyl-2′-deoxyuridine (Edu) staining also demonstrated that the proliferation rate of lnc*GDAR* overexpression cells was significantly increased compared with that of the control cells (Figure 7I). Furthermore, we found lnc*GDAR* overexpression significantly inhibited the expression of apoptosis-related mRNAs (*caspase-3*, *caspase-7* and *bax*) and significantly promoted the expression of anti-apoptosis-related mRNA (*bcl-2*) (Figure 7J). Compared with control cells, the expression of apoptosis-related mRNAs (*caspase-3*, *caspase-7* and *bax*) and anti-apoptosis-related mRNA (*bcl-2*) were up-regulated and down-regulated in lnc*GDAR* knockdown cells, respectively (Figure 7K). The Western blot assay also showed that knockdown lnc*GDAR* increased the protein levels of bax, caspase-3, caspase-7, cleaved caspase-3 and cleaved caspase-7 but decreased the protein levels of bcl-2 and bcl-9 (Figure 7L–N). Taken together, the data suggested that lnc*GDAR* can inhibit GC apoptosis through affecting mRNA and protein expression of apoptosis- (caspase-3, caspase-7, bcl-2, bcl-9 and bax) related makers.

## 3. Discussion

Nutrition is one of the most important environmental factors affecting reproductive performance in livestock [33]. Numerous studies have shown that short-term nutritional supplementation increased intrafollicular glucose concentrations and elevated ovulation rate [34,35,36,37]. This is the result of metabolites acting directly on the follicle as a signal to regulate folliculogenesis, as glucose transporters and specific receptors are present in the follicle [38,39,40]. GCs are follicular somatic cells and published studies have showed that GCs have a greater capacity to take up glucose than oocytes [13,14]. Therefore, the effect of glucose concentration in the follicular fluid on granulosa cells is critical for follicular development. In vitro studies have found that excessive glucose levels induce apoptosis in granulosa cells and oocytes [41,42]. In line with these in vitro studies, we found that proliferation of GCs increased with increasing glucose concentration up to 8.4 mM then decreased at the higher concentrations, and the 8.4 mM glucose concentration gave the highest readings for all concentrations. This indicates that glucose has an important effect on the proliferation and apoptosis of GCs and the formation of follicles. However, information regarding the role of lncRNAs in granulosa cell apoptosis is limited, and no studies have reported the role of lncRNAs in the glucose-induced apoptosis of GCs. 

Recently, with the development of next-generation high-throughput sequencing, multiple lncRNAs have been identified and been shown to play important roles in many endocrine and metabolic diseases [43,44,45]. Ovarian lncRNAs were first identified by RNA sequencing in human cumulus granulosa cells. This study showed the presence of 89 differentially expressed lncRNAs between compact cumulus granulosa cells and expanded cumulus cells, suggesting a role for lncRNAs in cumulus expansion [46]. Liu et al. found that inhibition of lncRNA PVT1 can inhibit the apoptosis of ovarian granulosa cells in PCOS patients [28]. In luteinized granulosa cells, lncRNA CCNL overexpression promoted apoptosis, reduced glucose transport capacity, and impaired mitochondrial function; CCNL may be involved in PCOS pathologies, such as follicular atresia and insulin resistance [27]. Glucose is primarily metabolized through the glycolytic pathway, providing substrates such as pyruvate for energy production in oocytes and follicular somatic cells [47]. A lncRNA responsive to energy stress has been discovered: lncRNA ZNF674-AS1, which can play an important regulatory role in GC proliferation, glycolysis and AMPK activation [48]. Consistent with these studies, our RNA-seq and bioinformatics analysis data on sheep GCs under different glucose treatments showed that the targeted genes of differential lncRNAs were significantly enriched in apoptosis and glucose-metabolism-related pathways, including the NF-kappa B, Jak-STAT signaling pathway, HIT type 1 signaling pathway, TNF signaling pathway, p53 pathway, apoptosis and glycolysis. This suggests that lncRNAs are involved in the regulation of granulosa cell growth by glucose. 

Granulosa cell apoptosis is a tightly controlled process that depends on the balance between anti- and pro-apoptotic factors [44]. In this study, we focused on a new lncRNA, lnc*GDAR*, by constructing a network of ceRNAs (lnc*GDAR*-miRNA-mRNA) around the apoptosis-related signaling pathway. We found that lnc*GDAR* may control follicle development by regulating GC apoptosis and proliferation. Compared with the control and high-glucose groups, the low-glucose group showed a trend of high expression of lnc*GDAR*; thus, this lncRNA may have a protective effect on the development of GCs. Next, we demonstrated that lnc*GDAR* inhibits the apoptosis of GCs by regulating the expression of apoptosis-related mRNAs and proteins (caspase-3, caspase-7, bcl-2, bcl-9 and bax), indicating that lnc*GDAR* controls follicular development by regulating the apoptosis of GCs. Further studies to investigate other functions of lnc*GDAR* in regulating GCs (e. g. steroidogenesis), follicular development, or ovulation in vivo and reproductive performance, such as litter size, in sheep are required. 

## 4. Materials and Methods

### 4.1. The GCs Model of Glucose Treatment

Fresh ewe ovaries (from thin-tailed Han sheep, ages ranged from 1 to 1.5 years) were collected at the local abattoir (Baoding, Hebei, China) and transported to the laboratory within 3 h in a buffered saline solution supplemented with streptomycin/penicillin mixture (1%) maintained at 37 °C [49]. Small immature follicles between 3 and 5 mm in diameter were punctured with a disposable syringe; follicular fluid was collected from a number of ovaries (ovaries number > 50) to negate any individual animal effects. The follicle suspensions were pooled and GCs were harvested immediately after centrifuging at 1000× *g* for 10 min. GCs were counted with a hemocytometer (Axio Vert. A1, Zeiss, Oberkochen, Germany) and their viability was confirmed by trypan blue exclusion. Then, the GCs were seeded in cell culture plates (Thermo Fisher Scientific, Waltham, MA, USA) at a density of 2 × 10^5^/well and cultured in Dulbecco’s Modified Eagle Medium (DMEM/F12, Gibco, Carlsbad, CA, USA) supplemented with 10% fetal bovine serum (FBS) (Gibco, Carlsbad, CA, USA) and 1% streptomycin/penicillin mixture in a humidified atmosphere at 37 °C and 5% CO_2_ for 48 h with the medium changed every 24 h.

Briefly, the GCs were seeded into different plates (1 × 10^6^ viable cells/well in 6-wells and 1 × 10^4^ viable cells/well in 96-wells) in culture medium (DMEM/F12 supplemented with 10% FBS and 1% streptomycin/penicillin mixture) at 37 °C in a humidified atmosphere containing 5% CO_2_ until the cells’ confluence reached up to 80%. Then the medium was removed to establish a model of GCs treated with different concentrations of glucose. All treatments were cultured without glucose and serum but containing streptomycin/penicillin mixture (1%) for 8 h; then, the treatments received various solutions of glucose and were cultured for an additional 24 h, as follows: 0 mM (i.e., zero glucose), 2.1 mM (378.3 μg/mL), 4.2 mM (756.6 μg/mL), 8.4 mM (1513.2 μg/mL), 16.8 mM (3026.4 μg/mL), 33.6 mM (6052.8 μg/mL). For each treatment, cells were allocated to six glucose-treated groups with different concentrations, each with three replicates. These doses were designed to span the normal physiological ranges of follicles (1.1–2.1 mM) [6,7,50,51]. The 33.6 mM group represents 30 times the physiological concentration of glucose in follicular fluid and was used to detect changes of steroid hormones at super-physiological concentrations. This culture system has been developed so that GCs retain hormonally responsive aromatase activity and do not luteinize with time in culture [52,53,54]. The media and GCs were collected for subsequent measurements after the 24 h treatment period. 

### 4.2. Cell Proliferation Assay

Cell proliferation was monitored using a TransDetect CCK (TransGen Biotech, Beijing, China) according to the manufacturer’s protocols. Absorbance was measured using a Model 680 Microplate Reader (Bio-Rad, Hercules, CA, USA) by optical density at a wavelength of 450 nm.

### 4.3. Cell Apoptosis Analysis

Cell apoptosis was analyzed by flow cytometry (BD Biosciences, NJ, USA) using an annexin V-fluorescein isothiocyanate/propidium iodide apoptosis detection kit (Vazyme, Nanjing, China). All data were analyzed using FlowJo software.

### 4.4. RNA Extraction, Complementary DNA (cDNA) Synthesis, and qRT-PCR

Total RNA was extracted from cultured cells according to the manufacturer’s instructions and supplied with the TRIzol Reagent (Invitrogen, Life Technologies, Carlsbad, CA, USA); cDNA synthesis for RNA (mRNA and lncRNA) was carried out using the PrimeScript RT Reagent Kit with gDNA Eraser (Perfect Real Time) (TaKaRa, Otsu, Japan). Primers of mRNAs and lncRNAs were designed and synthesized by GenechemBio (Shanghai, China). The specific quantitative primers for mRNAs and lncRNAs are listed in Tables S1 and S2, respectively. Real-time quantitative PCR reactions were performed on a Bio-Rad CFX96 Real-Time Detection System using an iTaq Universal SYBR Green Supermix Kit (Bio-Rad Laboratories Inc., Hercules, CA, USA). Data analyses were performed using the 2^ΔΔCt^ method as described previously. GAPDH and β-actin were used as internal controls for mRNAs and lncRNAs, respectively.

### 4.5. Western Blotting

Total proteins from tissues and cells were lysed conforming to the user’s guidebook of RIPA lysis buffer (Beyotime, China); this was followed by separation with 10% sodium dodecyl sulfate-polyacrylamide gel electrophoresis (SDS-PAGE) and transfer with polyvinylidene difluoride (PVDF) membranes (Bio-Rad, Hercules, CA, USA). After that, membranes were subjected to a standard blocking with 5% non-fat milk, hybridization with primary antibodies at 4 °C overnight, and incubation with secondary antibodies at room temperature for one hour. The bands were detected according to the instructions of the ECL detection kit (Santa Cruz Biotechnology, Santa Cruz, CA, USA). The primary antibodies are presented as follows: Bcl-2 (Bcl-2, 1:1000; ab32124, Abcam, Cambridge, UK), Bcl-9 (Bcl-9, 1:1000; ab37305, Abcam, Cambridge, UK), caspase-3 (caspase-3, 1:1000; ab13847, Abcam), caspase-7 (caspase-7, 1:1,000; ab255818, Abcam, Cambridge, UK), cleaved caspase-3 (cleaved caspase-3, 1:1000; #9661, Cell Signaling Technology, Boston, MA, USA), cleaved caspase-7 (cleaved caspase-7, 1:800; #8438, Cell Signaling Technology, Boston, MA, USA), bax (bax, 1:1000; ab32503, Abcam, Cambridge, UK), and goat anti-rabbit IgG (H + L) secondary antibody (1:5000; ab6721, Abcam, Cambridge, UK ).

### 4.6. RNA-Seq and Bioinformatics Analyses

#### 4.6.1. RNA Extraction, Library Construction, and RNA-seq

Ovine granulosa cells subjected to control (*n* = 3, 0 mM glucose groups), low groups (*n* = 3, add 8.4 mM glucose groups), and high groups (*n* = 3, add 33.6 mM glucose groups) were used for RNA-seq. Total RNA of each sample was isolated using Trizol reagent (Invitrogen, Carlsbad, CA, USA). RNA integrity was assessed using the RNA Nano 6000 Assay Kit of the Agilent Bioanalyzer 2100 System (Agilent Technologies, Santa Rosa, CA, USA). Ribosomal RNA (rRNA) was removed from the total RNA using the Ribo-Zero TM rRNA Removal Kit (Epicentre, Madison, Wisconsin, WI, USA). A total amount of 3 µg RNA per sample was used as input material for the RNA sample preparations. Sequencing libraries were generated using the NEBNext^®^ UltraTM RNA Library Prep Kit for Illumina^®^ (NEB, USA) following the manufacturer’s recommendations, and index codes were added to attribute sequences to each sample.

The whole-transcriptome libraries sequencing was performed by Novogene Bioinformatics Technology (Beijing, China). The clustering of the index-coded samples was performed on a cBot Cluster Generation System using TruSeq PE Cluster Kit v3-cBot-HS (Illumia) according to the manufacturer’s instructions. After cluster generation, high-throughput RNA-seq was performed on an Illumina Hiseq Xten platform (Illumina, San Diego, CA, USA), and 150 bp paired-end reads were generated according to Illumina’s protocol. Raw data was filtered for clean data. All the downstream analyses were based on the clean data with high quality. Clean reads were aligned using Hisat2 [55]. The mapped reads of each sample were assembled by Stringtie [56].

The CNCI (Coding-Non-Coding Index), CPC (Coding Potential Calculator) and PFAM (Protein Families database) were chosen as annotation references for coding potential analysis of lncRNA. Transcripts predicted with coding potential by either/all of the three tools above were filtered out, and those without coding potential were our candidate set of lncRNAs. HTSeq v0.6.0 was used to count the read numbers mapped to each gene. Fragments per kilobase million (FPKM) of each gene was then calculated based on the length of the gene and reads count mapped to this gene. [57]. Genes with an adjusted *p*-value < 0.05 and absolute value of |log (Fold change) |> 1 found by DESeq2 [58] were assigned as differentially expressed. Log (Fold change) was calculated based on standardized counts and has a strong correlation with FPKM value.

#### 4.6.2. Target Gene Prediction and Functional Annotation Analysis

Prediction of DE lncRNAs by cis-and trans-acting target genes. For each lncRNA locus, the 100 kb downstream and upstream protein-coding genes (without overlap) were firstly identified as cis-acting target genes. Then, the genes that overlapped with the lncRNAs predicted by Lnctar (http://www.cuilab.cn/lnctar, accessed on 10 November 2015) were selected as the trans-acting target genes.

Gene Ontology (GO) enrichment analysis of differentially expressed genes or lncRNA target genes were implemented by the GOseq R package, in which gene length bias was corrected [59]. GO terms with corrected *p* value less than 0.05 were considered significantly enriched by differential expressed genes. All the overlapping genes in each cluster were retrieved for data visualization using the RCircos package. We used KOBAS software to test the statistical enrichment of differential expression genes or lncRNA target genes in KEGG pathways [60]. Alter native splicing analysis alternative splicing events were classified to 12 basic types by the software Asprofile v1.0.

#### 4.6.3. Construction of lncRNA-miRNA-mRNA Interaction Network

To investigate lncRNA that may have functions in regulating apoptosis, we constructed a lncRNA-miRNA-gene interaction network belonging to apoptosis-related signaling pathways. It contained 15 differentially expressed apoptosis-related mRNAs, all of which can target a lncRNA (TCONS_00128966) through the ceRNA network.

Firstly, we predicted the potential target genes of differentially expressed miRNAs using TargetScan. Secondly, RNAhybrid, a tool for finding the minimum free energy hybridization of a long and a short RNA, was used to predict the target lncRNA of differentially expressed miRNA. As miRNA can repress the expression of mRNA, it can also inhibit the expression of its target lncRNAs. Therefore, the predicted target lncRNAs that have opposition expression patterns of their corresponding miRNAs were selected as candidate target lncRNAs for differentially expressed miRNAs; Finally, we constructed a lncRNA-miRNA-mRNA interaction network using Cytoscape 3.6.1 (https://cytoscape.org/, accessed on 11 september 2018). 

The raw sequencing dataset supporting the results of this study have been submitted to NCBI BioProject (PRJNA825818) (https://www.ncbi.nlm.nih.gov/geo/query/acc.cgi? acc=GSE200668, accessed on 12 April 2022).

### 4.7. Fluorescence in Situ Hybridization of lncRNA GDAR 

Fluorescence in situ hybridization (FISH) was performed using a fluorescent in situ hybridization kit from GenePharma (Shanghai, China), in accordance with the manufacturer’s instructions. We used U6 and 18s as our endogenous controls, which were obtained from GenePharma. The stained samples were imaged under confocal microscopy.

### 4.8. siRNA and Transfection

The granulosa cells were incubated overnight (at 60–70%, confluence); transfection or co-transfection were performed using Lipofectamine 2000 (Invitrogen, Shanghai, China) for 48 h. Quantities of 100 nM/mL siRNA-lnc*GDAR* and 1.25 mg/mL pcDNA3.1-lnc*GDAR* plasmids were used in this study. siRNA specifically targeting lnc*GDAR* or nonspecific control were synthesized by Gene Pharm. Their sequences were as follows: si-lnc*GDAR* (GCAGCACAGUUAUUAUAUAUG), si-NC (UUUUCCGAACGUGUCACGUTT). The lncRNA TCONS_00128966 sequence was amplified by PCR. The DNA sequence was verified and subcloned into the pCDNA3.1 vector, generating pCDNA3.1-lnc*GDAR*.

### 4.9. 5-Ethynyl-2′- Deoxyuridine (EdU) Assay

The GCs were seeded in 12-well plates (1× 10^6^ viable cells/well in 12-wells). When the cells grew to a density of 50% confluence, they were transfected with overexpression plasmids or siRNA. After transfection for 24 h, GCs were exposed to 50 μM EdU (RiboBio, China) for 2 h at 37 °C. Subsequently, the cells were fixed in 4% paraformaldehyde for 30 min, neutralized using 2 mg/mL glycine solution, and then permeabilized by adding 0.5% Triton X-100. A solution containing EdU (Apollo Reaction Cocktail; RiboBio, Suzhou, China) was added, and the cells were incubated at room temperature for 30 min. The nuclear stain Hoechst 33,342 was then added and incubation was continued for another 30 min. A fluorescence microscope (DMi8; Leica, German) was used to capture three randomly selected fields to visualize the number of EdU-stained cells.

### 4.10. Flow Cytometric Analysis

For the flow cytometry analysis of the cell cycle, GCs were seeded in 12-well plates. When the cells grew to a density of 50% confluence, they were transfected with overexpression plasmids or siRNA. After transfection for 24 h, the cells were collected and fixed overnight in 70% ethanol at 4 °C. Subsequently, the fixed cells were stained with a 50 μg/mL propidium iodide solution (Sigma Life Science, St. Louis, MO, USA) containing 10 μg/mL RNase A (Takara, Japan) and 0.2% (*v*/*v*) Triton X-100 (Sigma Life Science, St. Louis, MO, USA). This was then incubated in the dark and at 37 °C for 30 min. Flow cytometry analysis was performed on a BD Accuri C6 flow cytometer (BD Biosciences, San Jose, CA, USA), and data were processed using the FlowJo7.6 software (Stanford University, Stanford, CA, USA).

### 4.11. Caspase3/7 Activity Assay

Primary GCs were seeded in 96-well plates and following the different dose glucose treatment. After transfection for 24 h, GCs cells were subjected to the caspase 3/7 activity assay by Caspase-Glo_3/7 Assay Systems (Promega, #G8091) according to the manufacturer’s instructions. The assay was conducted in triplicates and repeated independently three times, which was represented as a fold increase in fluorescence calculated by comparing cells with untreated control cells.

### 4.12. Statistical Analysis 

Each experiment was performed in triplicate; data were analyzed by SPSS version 22.0 (SPSS Inc., Chicago, IL, USA). All data were normally distributed continuous variables and reported as the mean ± standard error of the mean (SEM). Statistical differences were determined by one-way analysis of variance (ANOVA). Tukey’s test was used to estimate the significance of the results. A *p* value < 0.05 was considered statistically significant.

## 5. Conclusions

In conclusion, numerous lncRNAs and mRNAs, corresponding to varying amounts in ovine GCs with different glucose treatments, were annotated. The lnc*GDAR*, was found to be predominantly expressed in the GCs with the low glucose group and involved in GC apoptosis. The findings confirmed the mechanism by which lnc*GDAR* regulates ovine granulosa cell apoptosis through affecting apoptosis-related gene expression. Therefore, this study identified a candidate lnc*GDAR* involved in ovine GCs differentiation, providing profound insights into the regulatory mechanisms of nutrients underlying follicular development and a basis for the development of new therapeutic strategies for reproductive diseases. 

## Figures and Tables

**Table 1:** The Detailed Information of RNA Sequencing.

Sample Name	Raw_reads ^1^	Clean_reads ^2^	Raw Bases(G)	Clean Bases(G)	Error Rate (%) ^3^	Q20(%) ^4^	Q30(%) ^5^	GC_Content(%) ^6^
CON_1	155,947,936	154,249,698	23.39	23.14	0.03	97.84	93.89	50.13
CON_2	129,736,352	128,235,276	19.46	19.24	0.03	97.75	93.65	51.77
CON_3	108,890,118	107,694,844	16.33	16.15	0.02	98.16	94.57	56.54
LOW_1	102,687,890	101,415,560	15.40	15.21	0.03	97.81	93.79	52.45
LOW_2	101,415,560	126,120,380	18.92	18.65	0.03	97.93	94.15	54.28
LOW_3	98,057,424	96,829,522	14.71	14.52	0.03	97.86	93.83	54.01
HIGH_1	144,402,918	141,776,652	21.66	21.27	0.03	97.68	93.59	55.14
HIGH_2	144,402,918	114,360,744	17.42	17.15	0.03	97.72	93.76	53.69
HIGH_3	107,442,946	106,281,570	16.12	15.94	0.02	98.15	94.54	56.11

^1^ Raw reads: the number of reads in the original data; ^2^ Clean reads: The number of reads after filtering the raw data; ^3^ Error rate: Overall sequencing error rate; ^4^ Q20: Percentage of bases with a mass value greater than or equal to 20; ^5^ Q30 (%): Percentage of bases with a mass value greater than or equal to 30; ^6^ GC (%): sample GC content.

## Data Availability

The datasets used during the current study are available from the corresponding author on reasonable request.

## Supplementary Materials

The following supporting information can be downloaded at: https://www.mdpi.com/article/10.3390/ijms23095183/s1.
