# Supplementary material for: Long Non-Coding RNA GDAR Regulates Ovine Granulosa Cells Apoptosis by Affecting the Expression of Apoptosis-Related Genes
Source: Int J Mol Sci. 2022 May 6;23(9):5183. doi: 10.3390/ijms23095183 (PMC9104640; doi:10.3390/ijms23095183)
Supplement: Supplementary file 1 [file ijms-23-05183-s001.zip › Supplementary Materials/Figure S1-3.pdf]

Figure S1. The significantly enriched GO terms of lncRNAs regarding cellular component. (A–C) GO categories (cellular component) of differential lncRNA target genes in different glucose treatment groups (CON versus LOW, CON versus HIGH and LOW versus HIGH).

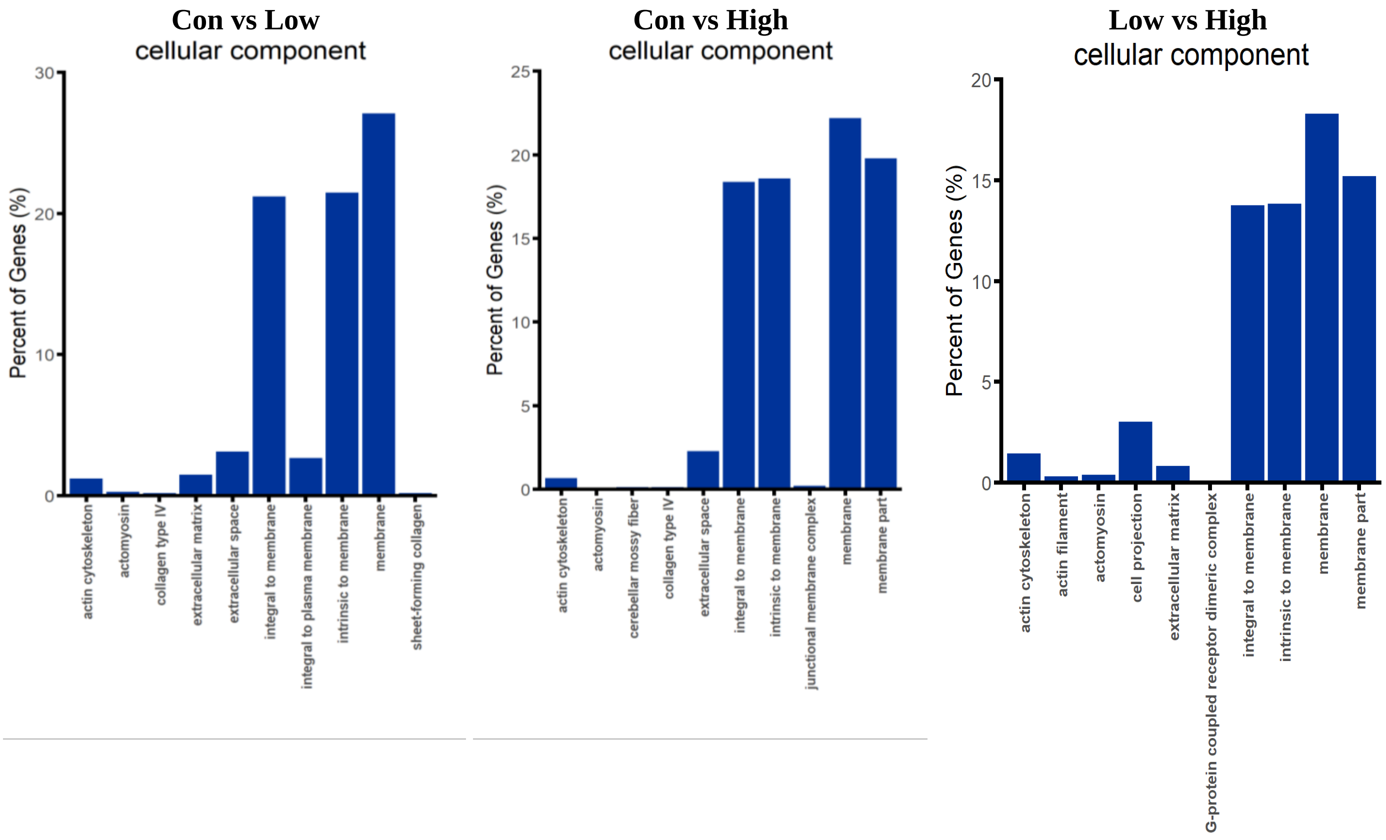

Figure S2. The significantly enriched GO terms of lncRNAs regarding molecular function. (A–C) GO categories (molecular function) of differential lncRNA target genes in different glucose treatment groups (CON versus LOW, CON versus HIGH and LOW versus HIGH).

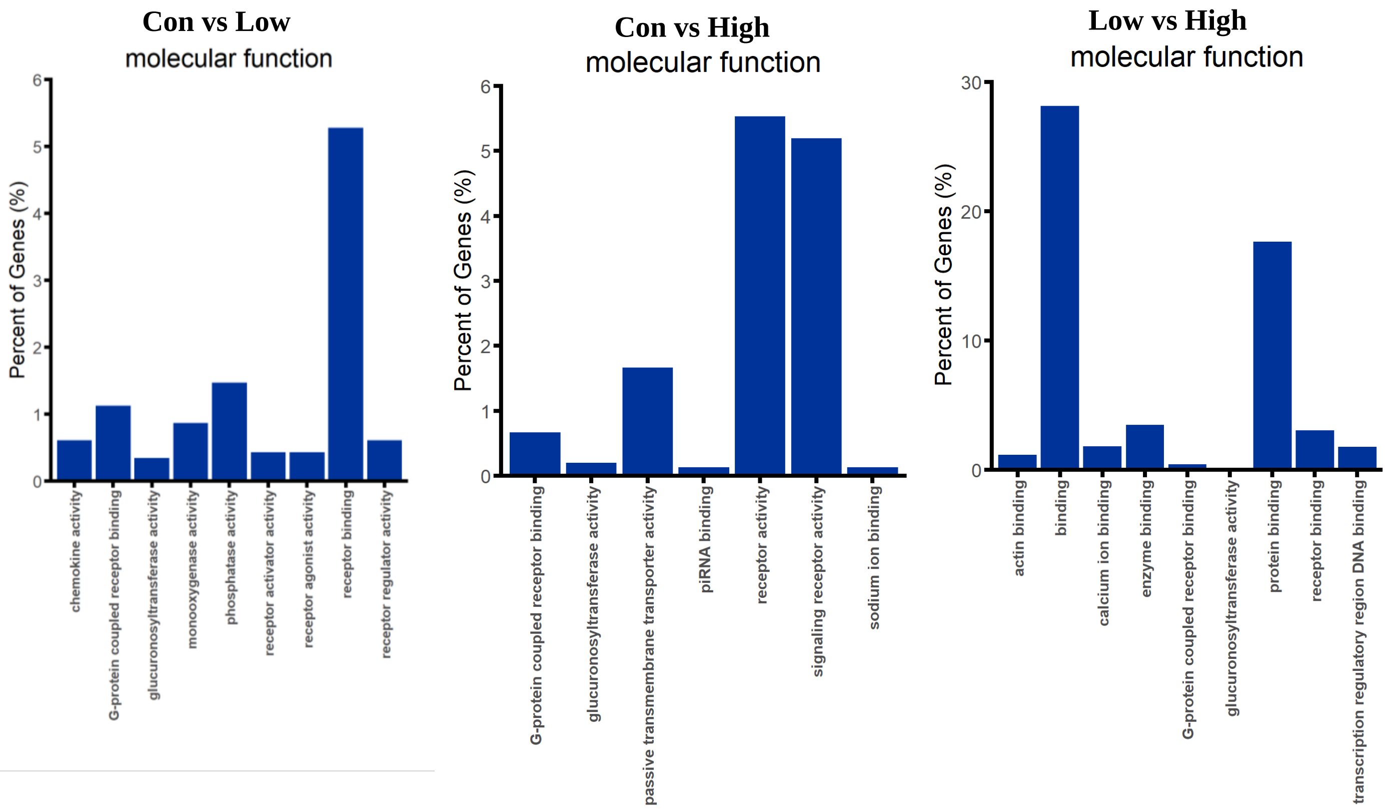

Figure S3. RNA sequence of TCONS_00128966

TATATATATATAATATGTCCACCCACTGAGTGGGCAGGCCTTGGATGCTCTCTGGGGGCCAGG
TCTCGCTGCCTGTATGCTCAGTCAGGTCTAACTCTTTGTGTCCCCATGGACTGTAACTCACC
AAGCTTCCTCTGTCCATGGAATTTTCCAGGGAAGAATATTGGAGTGGGTTGCCATTTCCTAC
TCCAGGGCATCTTCCTGACCCAGGGACTTAACGCCTCTCTCTTGCAAATCTCCTGCACCGGC
AGGCAGGACTCTACTAACTGTGCCACCTGGGAAGCACTGGGGGACCAGGGTTGGGGGGGG
GAGTGTTTATGGGGGAGGAATATCAAGCCAAGTAAGAAATTCCTGCCCACTGCAGTTCCTA
GGGGGCTAGAGTTCAGTGTGGAATTGTCTGTGGAATTCTCCAGGCAAGAATACTGGAGTGG
GTAGACATTCCTTTCTCCGGAGGAATCTTCCCAACCCAGGGATCAAACCCAGGTCCTCCTAT
ATTGCAAGCAGATGCTTTACTGTCTGAGCCACAAAGGAAGCTTCAGAGTTCAGTGTGGAAC
TGCAGAAGCAGATATTTCTATTATGACTGTGATGAGCCTGTCAGGGGGGGTGATGAGCCGA
GCCCGCGGGTGTTCCTGAAGCCAGCCTCCTTTGTGTTGCCTGCCCGGGGCTACTACACGTT
GGGGGGTGGGGGCAACTCCCGGCCACGCCCTTCCTCACCTTTGTCTTGAGCCACATTGTTC
TGCGCCTGTGGACCCTCATCAGCTGTTCCTGGCCCTCACACCCCAGTTCCGTTGTTTCTTCC
CCACGTTTTCCCACTATGCCCCAGTTGCCTTGCACACCTCTGCTTCTTCCTATAGCAAATGCC
TAAGGATGATCACAGCTCACTTTCACAGGCTAAGCCACAGTTTCACAACCCTGTGGAGTGG
GTCTAATATTATCCCCATTTCACAGGTGAGGAAACCAAGGCCAAGAGATTAAGTATGTAGCA
TAAGGCCACAGTGGGGAAGGAAATAGCAATCCACTCCAGTATTCTTCCCTAAGGCCACAGA
GCTAGGAGGTGATGGGCCTGGGGTTGAACCCGGGCAGTCTGGTTCCAGTCTGTCCTAGTAA
CCACTGCACTAGGTTGACCTCTTAACCCGTCATTTTCTTTCCAGCTCTCAAACCACATGTTC
ATTCCCAGGCTTCCACACAGTAGGTGCCCATTAGAGGTTTGACAGATGAGTTTCGTCTTCCT
ATCCTGCCTGCCTCGGCGTCACGCCTTGTACATTTGCCATTAATAAAGAAATGAATACGTGG
GCTGACGAACAGACGAATAGATGATGCCACCTGTTGGTGTGTGTGGAAGCTGTAGTTACCA
GCATTCTTCAGGGCAGAGGTGCAAGTTCGTAATGACTGGACAGGTTCTGAAATCATCCCCG
TCGTTGGTGAATGCAGTTTTGAAATAACGTTGTCACTATAATGTCCCTAGCCAACAGGAGGT
AGTGTTTTTACTCTTCTATCAAAAGCCAAAGCCTCTTGGGGCATGGGACCTCCAAGGCTTG
GGCTGGGGCTCTTATTTAAAAGCGAGGAGGAGAGTGCTTCCCCAGGCAGGTAGTTAGGAA
GCCCATCCAGGGGTACTTGGAGATCAGAGCCAGCTTTTGGAGGATGAAAATGAGTTAAAAG
TCTGGCCATGATAATTCCCTTATCTCTGACTTCCTGCCAGCACTCCTGACGTCTGAGCTGCTC
AGAATCTGGGTGGCCTCTCTGCAGGGCTCTTGATGCAAAATGAGCCCCAGGTGTTGGGGAT
AAAAAACCCACTGTTGGTCATTACCACGCCCCCTCTTCTGGGCCACCCTGGGTGCCCTGGC
TTTGTGGGGTTTTCCCCTCCAGCTCTGCTGATCTGCAGGCATATTTATTTGTAAGTGGAATTT
TTCAAAGGGAAGCACTTTACAAATGATGAAAAGAAAACAACTTGGGGTAAATGGCTCATCT
GGCAATGGGAAACAGCCCCTGGCATCCAGAGCTCTTTAACCTTGTCTGAACAGGCAGCAG
GGAACTTGGCTTCTGTATCATCTTACAGAAAATTTTTCTGTCTCCCTACTGAGGCCAGACCC
TGGGGAGAAAGAGCGGAAAACCCAACCCAGGACCCTTGGAAAATTTGATAGGTGGGTGTC
TCTCTACCCCATACGGTCAACCTGAGTGCCCTTCCTATCCAAGCCTCTGTGACTTCTGTCCC
CAGATGATTCCATAGTTCTCTGCTTGTCTCCTGACTTCTGCCCCAGTCACTTCTGATGCTGCG
GCCCACAGAAAGATCTAAAAATCAGAGTGGATCTGCTCCATTCCATCTGTGAACGAGGCCT
CTGGTGCCCCGTGCACCCGCATAGAAGCCGCAGAATTAAAGCTGGTCTTACCAAGGCCTGC
AGAGCCCCATGTGATCGGGCCCTTCTGTCTTTCTGGCCCCATTTCCTGCTGCAGCCACACTG
GCCTCTTTGCAGTTCTCAGTTCTTCAAGCCTGTTCCTACCCCAGGGCCTTTGCATCTGCCAT
CCCCTCTGCCTGCCTCCCCACATCCTCCTGGTTCTCCCTCATTTGGCTGTGGGCTGTGCTCA
GATAGCATCTTATCAATTGGGCCCTCCCTGTGGCCTTCTATAAAGTATCCCCCCTTCTCTCTT
GCCTGCACTCCCTGCCCCACCACCGTCAGCTTTAGGCTTGCTCCACAGTTATGGAGAAGGC
ACTGGCACCCCACTCCAGTACTCTTGCCTGGAAAACCCCATGGACGGAGGAGCCTGGTAG
GCTGCAGTCCATGGGTTCGCTGAGCATTGGATACGACTGAGCGACTTCCCTTTCACTTTTCA
CTTTCATGCACTGGAGAAGGAAATGGCAACCCACTCCAGTGTTCTTGCCTGGAGAATCCCA
CGGATGGAGGAGCCTGGTGGGCTGCCGTCTATGGGGTCGCACAGAGTCGGACACGACTGA
AGCGACTTAGCAGCAGCAGCACAGTTATTATATATGTTATTTCTATGTTCAGCTTTTTATAGTT
ATTTATATGTTCAGTTTCTGTCCTGTTACATCAAAATATACATCCGAATGTAAATGCCTTAAGG
ACAAGGACTTTATTTACTTTTGCTTTAGATACTTCTTTTTAGGGGGCACATCACATATGGTCT
ATCCATTCATCCTTTGATGGACATTTGGATGTTTTCCACCTTTTCACTGTTACAAATAATGCT
GCTGCAAACTTTTGTGTACAAGTGTCATGTGAATGAGGAAAGCTTTGGTTGGACATCTTCA
AGTCAGTTCACAGATGCTGTCTTTGAGGGGTATTCATGCCTGGAGCCATGTCCCAAATCTCC
CAGTGGCTCCTGAACTCATTGGTTTCAAATTATGATTGTATTTTTAGAGAGCTGTGTTACTTA
TCCCAGCAGGCATTCTTTTGCTATAAGACTGATGTATTATTACTGTTTCCACTCTCTGTGTGA
TTGGCTGGGTATCCCTTTAATGCTGCCACCTGCAATTCCTTTCTTTGTACAGACTAGTTAACA
TCTTATTTGTTTGTTTATTTTTATTTTGACTGTGCTGGGTCTTTGTCCTAAAGCTCAGTCTTCT
CTAGTTGTGGCCCTTGGGCTTAGTTGTAGGATCTTATTTCCCTGACCAGGGATTGAACCTGA
GTCCCCTGCATTGGCAGGTGAATTCTTGACCACTGGACCACAGAGAAGTCCCCTAGTTACC
CACCTGATGTCTCTTCCAGTTTTACTCTGCTCAGCTATGTGGCTTCATTTGACTCCATCCTCA
GAACTGATTGTGTTCCTTTCTCCCTAGAGCTTCTCTTTTCTGAATGAAATTTGCCATCCTGCA
CCAAGAACCCTCGACTCTAAGTGGATTTGTCTGAAACGTGATCTTTGAGTTCGTCTCATTCC
AGTCAACTTCTTTATCTCCTAATTGCATTACCCACGACCCCAGTAGAATTCCAAAAAGCATT
TATAGAAGATGCATTAAGTTTGGATTGCTGGTGAGGTTGTGATGACAAAGGCAGCCGCAGG
GCTGCTCTGCCAAGTGCCAGGGTTTGAACTCAGGGTAGCTTTTCTCCTTTGCCCGAAGGAA
AGCATGGGCAGGTTTGCTCTGCAGGGCATCTGTAGCAGCAGCCAATCTTCTTGGAAAGGAG
AACTGAGGTGCAAAAGGCCAGCTCTTTCATTCTGCTGTGAGGACACAAAGGAGGGTTTGT

TGAAAATATCTGTCAAAGGTGGGTGGGATTCCAGAGATCATCCATGTCTCACGTGATCAGAGCACC
TTTTTAAAGATGAATGAATGAATTGATGCTTTTCTTTTTGGCTGCACTGGGTCTAAGTTACTGCATGT
GAGCTTTCTCCAGTTACGGCAAACGAGGGCTACTGGCTAGTTGCAGTGTGCGGGCTGCTGCTTACA
GTGGCTTCTCTTGCTGAGGAGCACGGGCGCTAGGCATGAGGAGCATGAGGAGCTAGGCACACTGG
CTTTGGGAGTTGTGGCTCACAGGCTCAGTTAGCTGCCTGCAGGCAAGTGGGGTCTTCCACAATCA
GGGATCAAACAAGTAGTGTCCCCTGCCTTGCAAGGTGAATTCTTAACCACTGACCCACTAGAGAA
GCCCCTGTCCAGAGCACTTTTGAGTGCGTTTTCCTGCGTGTAGATCAAACCTCAGGGCCTTTGCAC
GTGCGCTTCCGTGTCACTCAGGACTCTGTTCCTCTGTCGCCACATTGGGAAGGTCTTCCCTGTACA
GCCTGCCCCCACCACTCACCTCCCACCTTCACTCTTGGCCCCCTTCCTGGGCTTTCTTTTTCTTTAC
AGCACTTATCTTCACTTTGTATATTATCTATTTCTTTGTCTCTCGCTGTTCCTCAACAGAACGCAAGC
CCCCTGAGAGTGCATCAGCCAGTGTTACATCTTTAAAACTTAACACGGTTCTCGGAACACAGGAG
GGACTCAGTGTGTGTTGAGTGAATGCATGAATGCATTTGCTTTTTATGAAAACCCTTGGGGCAAAG
GTACTGTTAATGCCGTCATGTGGGAGTAGGAACCAGAGTCAGATGACTGAAGACAATTACTCAAG
GTCACAAGCTGGGATGTGACTGAGCCCTGAATTGAGTTGGCTCTTCTGGGTCCCTGGACAGCCAC
CTCCCAGGCTTACAGAGCCCAAGAGAGCCTCTGTAGGTCCTTTGGAAATTCTCAGAAAAGGCCAC
AGTCTTCAGCAGGAAGTGGCACACGAGGTGCACAGAATGGAGTGGAAATTTTAAGAGCTATGGAG
CAAGGATTTCCTGTGACCTGAGATGGAAAACAGGAAATAAATGAAACCTGGAAAAACAGTGGTG
GAAGAGACTTGCAGTGATCAGGGAGGGCGGCAGTGGAGGGAGGGAGGGTGACGCAATGGAAGT
GGCTGCAGGAGGTCGTAAACCCGGAGGAATCCCTCCACAGGATGTCTTAGGAGCCTGTGATGGAC
GCAGAGCCCACTGGTCCCCTCAGGCTGTTCCGTGCATTTCCCCCCACCCCTAGTATGCCCAGATGT
CTCTCTGCAGAGGATTTAGCAGTCCAGAGGAGGAGGGGCAGTACCCTGCGACCATGAACTCTTGA
GTGCTGTGCTCAGGAGAAACCACAGATACCCCAGGTCCCACTGGATCTGACTGTGGGGTGCGTGC
TGCGACTTTTCTGTGGGAGGCAGTACTTGTATGGATGGGGAATCAGGGTGTCTTCCATTTGTGAAA
ACAAGTTGGCAGAAAAAGGAAGCCGGTGGTGGCGCCAGAAACCTCACTCAGGATTATCAACTTG
GCTAGTACTGGTGGTCACAGAGCATCAGGTGCTGATGTGAGGAGGTCAGGACTGGAATCCAGAGG
CTCCCGGGCCACCTCCTGTTCCTGATCCTGGGCTTGCTTTCCGTGCATGTTTTAACGCAGCCTCAAG
TCCTGCCTGTAGACTTTCCTACCTGTTCCGTGCCGGCTCTCCAGAGACCCACAGCCAGAACCTCCT
CCGAGAGGCTCCGGGGGACTTGTCCTTGGCAACAGCACACAGACCACATGTGCTGTGGGGCTGA
GGGCTTTCAGAAATTTCGGGGGAGAGAGAGGTGACTGCTAAGCCCAGTGCATCAGAGCACAGGA
GGGGGAGAGTGGCCTGGGGATTGAAGAGAAAGCATGTGAGGACCCCCGCCCCCATCTCTGCACCT
GGGGCCCCAAGCCAGGCCTCGCCCTTAGGGTCCTCAGAAAGAGGCCCTTAGCCCCATGGGTGGGT
GGACGCCCCAGGGTGAGTTAGAAGCCCGGGATGCCGCTCGAATTCCCAGACTCTGCGTGGGACTG
AAGAAGGACTTTTAAAACTAGAGCTGACAGGTCAAAATAAACGCCCTCCATCGTGAGGAGCAGCC
GCATTTGGGCCTCTGCGGCTGCCTCTCTAACAACACCCTTCATTCAGTGTCCGCTTCATGACCCACG
CCTGGCTCCACGCAGCTCTCCTCACCGTGCTCCCCCGCTCTTGCCAGCGGCCTCCTTCCCGTCTCA
CCGGCCTGGCCTTGTGACTGGAGTGGCCAAGACTCGCCTTAGGGTGCGCCCTGCGGAGACGCAGC
AAGGGAGACAGGGTTCTTGTCTGAAGGCGCCCTCGGGCTGGGGGAGTTAGTGGGAGCATGTGGG
AGTGTGTGTGTGAGTCTGTATGTGAGTGTAAGTCTGTGTGTGAGTGTGTACATGATTGTGTATGTGA
GTCTATATGTGAGTGTAAGTCTGTGTGTGAGTGTG
